# On the hunt for B-cell lymphoblastic leukemia-initiating stem cells

**DOI:** 10.18632/oncotarget.22578

**Published:** 2017-11-21

**Authors:** Bartosch Wojcik, Fabian Lang, Michael A. Rieger

**Affiliations:** Michael A. Rieger: Department of Medicine, Hematology/Oncology, Goethe University Hospital, Theodor-Stern-Kai, Frankfurt am Main, Germany; German Cancer Consortium, Heidelberg, Germany; German Cancer Research Center, Heidelberg, Germany

**Keywords:** cancer stem cells, leukemia, surface marker reversion, plasticity, ALL

The view that tumors consist of a homogenous mass of clonal derived cells has dramatically changed in recent years. Tumors harbor an enormous heterogeneity of cells with distinct capabilities and functions. The heterogeneity originates from a differentiation hierarchy of tumor cells, similar to normal tissue organization of stem-cell driven organs, but also from clonal succession of subpopulations by randomly acquired genetic mutations and epigenetic changes. Both scenarios are certainly not mutually exclusive, and also stem and progenitor cells underlie mutational selection. Intratumoral heterogeneity is a major challenge for cancer treatment and disease monitoring. Functional studies revealed that not all tumor cells have the same ability to initiate tumor growth upon transplantation in receptive animal models. The tumor-initiating cells (TICs) were called cancer stem cells due to their similarities to normal tissue stem cells in their molecular and functional properties. They can renew themselves long-term and give rise to tumor cells lacking cancer stem cell properties. However, it is worth stressing here that TICs do not necessarily originate from stem cells, but may have regained stem cell properties. TICs caught major attention since they may provide important steps in the progression of malignant diseases, such as epithelial-to-mesenchymal transition, dissemination, long-term persistence, therapy resistance, and relapse of the disease. The prospective identification of TICs using distinct surface markers would allow their molecular and functional characterization, the design of detection methods for diagnosis and prognosis, and the development of targeted therapies against these detrimental cells. While functional evidence for the existence of TICs were provided for many tumor entities, their marker profile still remains largely undefined and controversial.

The best studied cancer in respect to TICs is acute myeloid leukemia (AML), where several reports describe an enrichment of leukemia-initiating cells (LICs) in the CD34+ CD38- lineage marker (Lin)- AML cell fraction [1]. Noteworthy, also in AML cases with no CD34 surface expression on the leukemic cells (about 25% of AMLs), functional LICs exist, and in CD34+ AML, LICs can also be found in CD34+ CD38+ and the CD34- cell fractions, albeit with lower frequency [1]. Due to these results, scientists extensively used CD34 and CD38 to enrich for LICs in B-cell precursor-acute lymphoblastic leukemia (B-ALL), however, not leading to a robust procedure in the field and leaving many controversies behind [2]. B-ALL is an aggressive malignancy of bone marrow precursor cells of the B-cell lineage, which still suffers from a poor prognosis, especially at relapsed and refractory stage.

We recently showed that the expression of the surface markers CD34 and CD38 are highly dynamically regulated on individual leukemic cells in B-ALL [3]. Prospectively enriched marker-defined subpopulations based on these two markers reverted back to the original heterogenous marker-expressing composition of cells. We evaluated this phenomenon at population, clonal, and single cell level, by which we were able to demonstrate that on individual cells CD34 and CD38 are dynamically expressed even within one cell generation. These observations only became visible by the continuous long-term tracking of individual cells and their progeny using time-lapse microscopy [3]. These results may explain some of the controversies in the usage of CD34 and CD38 for the enrichment of LICs in B-ALL. Another example of a plastic surface marker expression in B-ALL was given for the expression of CD19. CD19 was supposed to be present on LICs in B-ALL, however, it was shown that CD19 negative cells could re-express CD19, and vice versa, and that CD19 did not segregate leukemia-initiation capacity [4]. A plastic surface marker profile seems not to be an exclusive phenomenon for B-ALL: In ovarian cancer, potential cancer stem cell markers CD24 and CD44 showed a similar dynamic expression [5].

Whether the surface marker plasticity is linked to a functional plasticity requires further evaluation. Recently, it became evident that in B-ALL there is functional plasticity in non-LICs converting into LICs suggesting that the elimination of a common LIC population may not be sufficient for long-term disease management. Jeremias and colleagues showed in B-ALL that a transient quiescent cell cycle stage is linked to cells that can reinitiate the disease, resembling a behavior of normal blood stem cells, but not further differentiated cells [6]. Interestingly, proliferating cells gave rise to quiescent cells and vice versa, suggesting that this reversible mechanism could be linked to therapy escape mechanisms and relapse in B-ALL. Therefore, cellular plasticity in B-ALL raises high interest and has enormous clinical impact.

An even worse scenario is envisioned by the possibility that the majority of B-ALL cells may have LIC activity and are able to propagate the disease. Vormoor and colleagues could show in a few tested B-ALL cases that the majority of subclones are able to contribute to leukemia induction and progression. Genetic barcoding and deep sequencing impressively revealed that leukemia-initiating clones in B-ALL are abundant and equipotent in the formation of the disease [7]. In contrast, our work allowed us to isolate isogenic clones of the same ALL patient with profound functional differences in their leukemogenic potential [3]. The molecular profiling of these differential leukemogenic clones may reveal the leukemogenic mechanism and enlighten molecular targets which may lead to new therapeutic approaches and robust biomarkers. Many fundamental questions remain to be answered concerning cells with LIC ability in B-ALL, whether they can be discriminated and how plastic they are. Future clonal tracking approaches will enable to further elucidate evolving questions in this field. For example, if we assume a stochastic model with the majority of B-ALL cells harboring intrinsic leukemia-initiating capacity, how dependent are these cells on their environment or can they even shape their own niche? Are there clones which have a preference to home in distinct organs e.g. the bone marrow or the spleen? Do they engraft and expand in locally restricted areas, or do they disseminate immediately? Are treatment responses on subclones dependent on their subclonal location? Are there functional differences between B-ALL cells from patients with good treatment response and refractory patients? These questions will remain to be answered by expanding clonal and single cell studies in the future. Our unique ability of culturing patient-derived B-ALL cells will contribute to address a variety of these questions to reconstruct a more complete view of the subclonal architecture and cellular complexity in B-ALL [8]. The clinical need to eradicate LICs for efficient cancer treatment is apparent, whether these are rare cells or the majority of the tumor, however, in order to do so, we need to know our enemy first.
